# Supplementary material for: Majorana zero modes in impurity-assisted vortex of LiFeAs superconductor
Source: Nat Commun. 2021 Jul 6;12:4146. doi: 10.1038/s41467-021-24372-6 (PMC8260634; doi:10.1038/s41467-021-24372-6)
Supplement: Supplementary file 1 — Supplementary Information [file 41467_2021_24372_MOESM1_ESM.pdf]

Supplementary Information for

Majorana zero modes in impurity-assisted vortex of LiFeAs superconductor

Lingyuan Kong†, Lu Cao†, Shiyu Zhu†, Michał Papaj†, Guangyang Dai, Geng Li, Peng Fan, Wenyao Liu, Fazhi Yang, Xiancheng Wang, Shixuan Du, Changqing Jin, Liang Fu, Hong-Jun Gao* and Hong Ding*

†These authors contributed equally to this work

*Correspondence to: dingh@iphy.ac.cn, hjgao@iphy.ac.cn

Supplementary Figure 1 ~ Figure 13

Supplementary Table 1 and Table 2

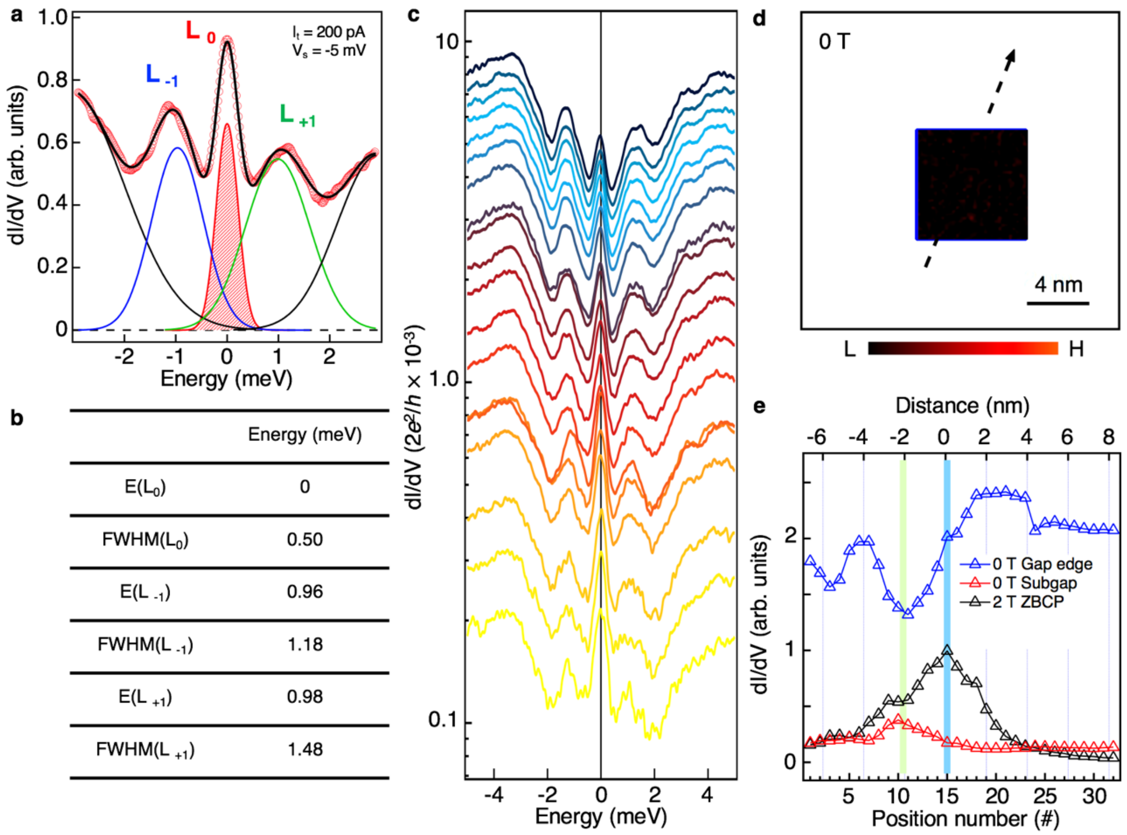

Supplementary Figure 1. Spectroscopic details of an impurity-assisted vortex. a, Multi-peak Gaussian fit of the *dI/dV* spectrum measured at the center of the impV shown in Fig. 3. **b,** Fitting parameters from **a**. **c,** Tunneling barrier strength evolution of **a**. The ZBCPs are stable at zero energy for over two orders of magnitude of tunneling barrier conductance. **d,** ZBC map on the area enclosed by the blue box indicated in Figs. 3a and 3b under zero field. It shows that the impurity does not introduce zero-energy impurity bound states in this area. **e,** Line profiles measured along the line indicated in **d** and Figs. 3a and 3b, which demonstrates the mismatch between vortex center (blue bar) and impurity center (green bar). The blue curve is the coherence peak line profile of the integrated intensity of *dI/dV* from -6 meV to -4.5 meV under zero field. The red curve is the subgap line profile of the integrated intensity of *dI/dV* from -1.8 meV to 1.8 meV under the zero field. The black curve is a replot of the zero-bias *dI/dV* intensity line profile under 2.0 T as shown in Fig. 3g.

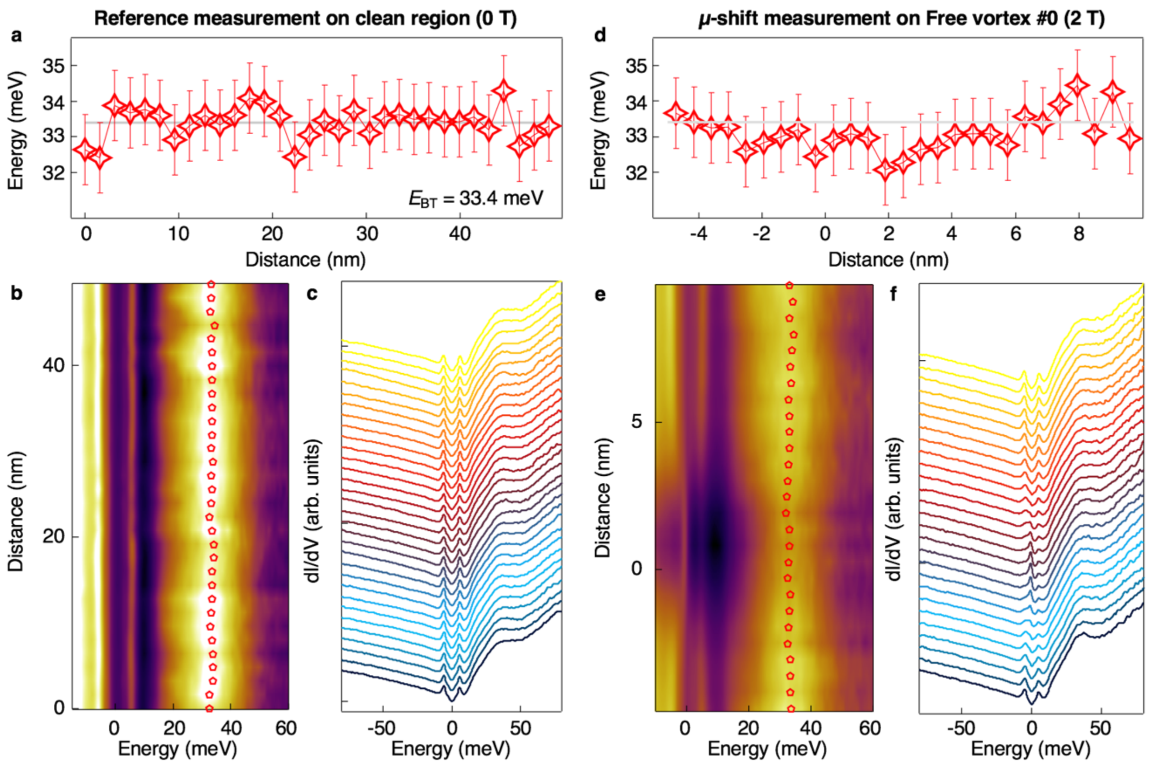

Supplementary Figure 2. Reference energy of band top in a clean surface region. a, Spatial evolution of band top energy across a long distance in a clean region without impurities and under zero field. The extraction method used here is introduced in Figs. 5f and 5g. **b,** Intensity plot with background subtracted. The red symbols are the same points as shown in **a**. **c,** Waterfall-like plot of raw data without linear background subtraction. **d-f,** Same as **a-c** respectively, but measured across a freeV and under 2.0 T. The extracted reference band top energy (E_{BT}) is about 33.4 meV, the error bar (1 meV) is determined by the spatial deviation of the singularity energy in a clean surface region.

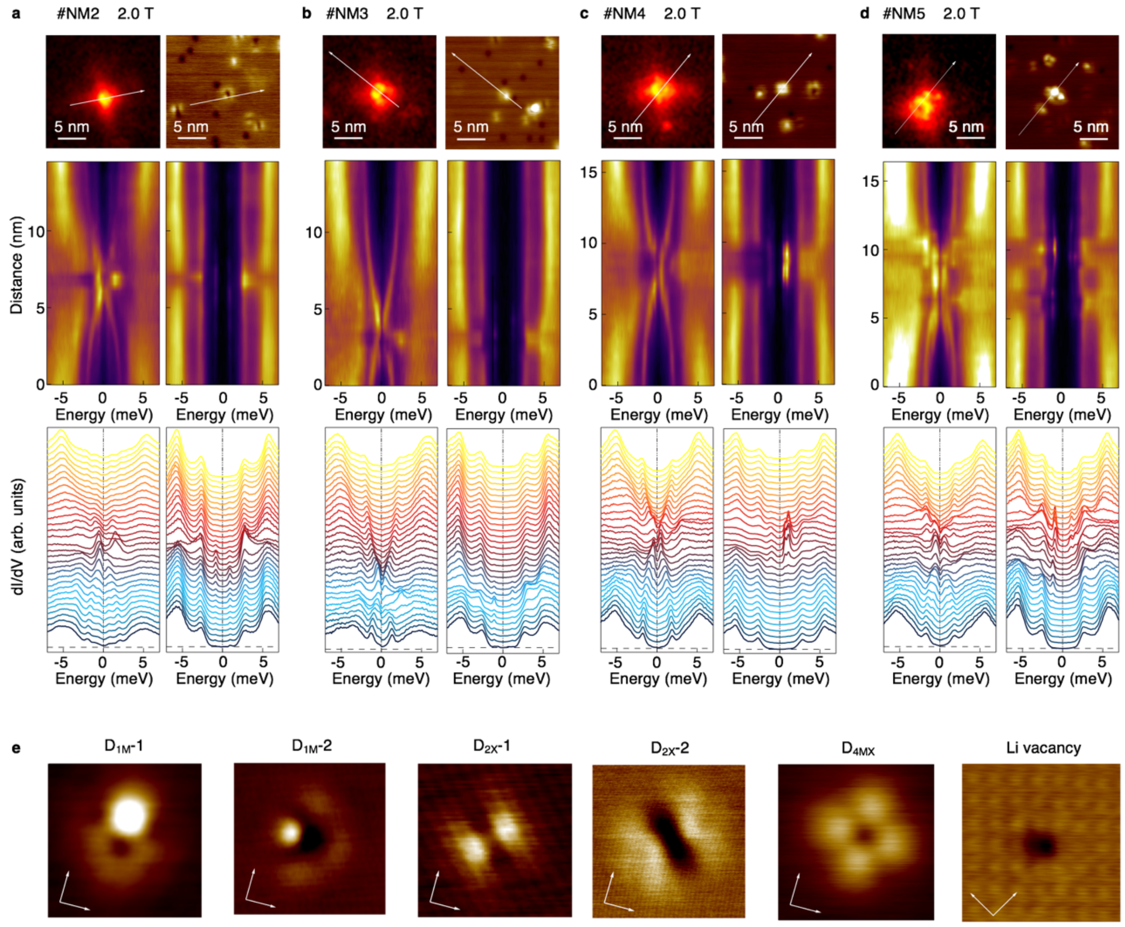

Supplementary Figure 3. Detailed characteristics of ordinary impVs coupled to weak impurities. a-d, In each panel, the first row shows the zero-bias vortex mapping under magnetic field (left) and the corresponding topography (right); the second row is the line-cut intensity plot measured with (left) and without (right) magnetic field; the third row are the corresponding *dI*/*dV* spectra of the second row. **e,** Typical weak impurities on LiFeAs surface. The arrows indicate the nearest Fe-Fe directions. Scanning area: 3 nm by 3 nm. The tags marked at the top indicate the symmetry of each impurity, and the letters in the subscripts of tags indicate the direction of symmetry axis, *e.g.* D_{4MX} stands for a D_4 symmetric impurity with its symmetry axis along Γ-X and Γ-M.

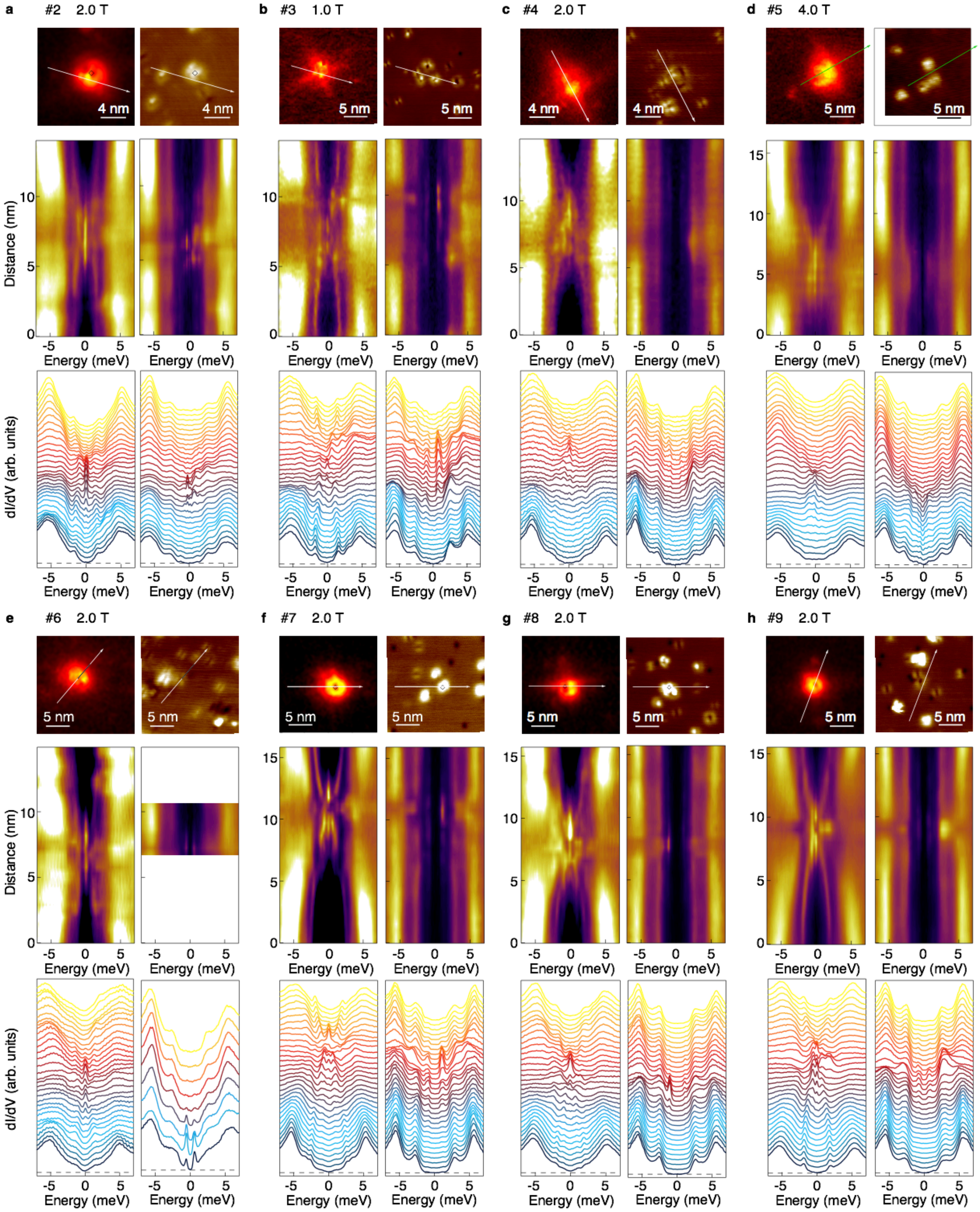

Supplementary Figure 4. Detailed characteristics of Majorana impVs coupled to strong impurities. In each panel, the first row shows the zero-bias vortex mapping under magnetic field (left) and the corresponding topography (right); the second row is the line-cut intensity plot measured with (left) and without (right) magnetic field; the third row are the corresponding *dI*/*dV* spectra of the second row. The impV #9 is the same vortex as studied in Fig. 5.

Supplementary Table 1. List of parametrs of nine Majorana impVs. The impVs #2 to #9 are shown in Supplementary Fig. 4, while #1 is shown in Fig. 3. E_{IBS} is the energy of the lowest impurity bound state. E_{VBS} is the energy of the lowest non-zero vortex bound states (E_{VBS}) of Majorana impVs (E_{VBS} is also the topological gap which protects MZM); μ-shift is the chemical potential around the impVs deviating from the reference value in a clean region. Positive value of μ-shift stands for electron doping.

	E_{VBS} (meV)	E_{IBS} (meV)	μ-shift (meV)
**#1**	0.92	0.48	Not measured
**#2**	0.87	0.24	Not measured
**#3**	0.58	0.78	Not measured
**#4**	0.48	0.8	Not measured
**#5**	0.72	0.44	Not measured
**#6**	0.79	0.55	+7.5
**#7**	0.56	0.83	+4.3
**#8**	0.63	0.94	+2.9
**#9**	0.61	0.7	+4.5

Supplementary Table 2. List of parametrs of five ordinary impVs. The impVs #NM2 to #NM5 are shown in Supplementary Fig. 3, while #NM1 is shown in Fig. 7. The impurity type is defined in Supplementary Fig. 3e.

	Impurity type	E_{IBS} (meV)	μ-shift (meV)
**#NM1**	D_{2X}	2.8	~+2
**#NM2**	D_{1M}-2	0.8	+2.7
**#NM3**	D_{1M}-1	1.1	+4.4
**#NM4**	D_{4MX}	0.56	+5.2
**#NM5**	D_{1M}-1	0.94	+3.9
